# P272: Reduction of complications associated with surgery by the implementation healthcare associated infections in the hospitals of the department of Borgou (Benin)

**DOI:** 10.1186/2047-2994-2-S1-P272

**Published:** 2013-06-20

**Authors:** C Degan, A Santos, P Hounye

**Affiliations:** 1 Health Ministry of Benin, Cotonou, Benin

## Introduction

Healthcare associated infections exact a heavy toll in terms of preventable death and disability in patients and are responsible for additional costs of hospitalization in health care facilities in resource-limited countries. The baseline mean attack rates for surgical site infections ranged from 27.3% to 54.2% in the surgical wards of four district hospitals in northern Benin. To this end, a program of prevention and reduction of surgical site infections is implemented to ensure the safety of patient care.

## Objectives

Halve the surgical site infections in hospitals targeted by the program.

## Methods

The clinical audit methodology is based on the use of standard criteria for prevention of surgical site infections in order to identify gaps and propose a plan for improvement. In addition samples of pus were analyzed in the lab to confirm the surgical site infection by identifying the causative organisms. A study protocol sheet with data collection monitoring has been developed for the calculation of incidence of suppuration with confidence intervals.

## Results

The observance of protocol implementation of preventive measures (hand hygiene, skin preparation, sanitation of the environment block ...) at all operating system has improved the following indicators:

- The rate of handwashing compliance improved from 25% to 45% in one year,

- The reduction of surgical site infections in hospitals by 25% in 2005 to 3% in 2012,

- The decrease in the average length of stay in health care for 22 days to 7 days.

## Conclusion

The introduction of the prevention of healthcare associated infections in the management of hospital management has reduced the risk of infection and improves the quality of patient care.

The change introduced in improving patient safety in these institutions remained a source of motivation for the generalization of the program in other services.

## Competing interests

None declared

